# Antitumor Activities of Human Placenta-Derived Mesenchymal Stem Cells Expressing Endostatin on Ovarian Cancer

**DOI:** 10.1371/journal.pone.0039119

**Published:** 2012-07-24

**Authors:** Lan Zheng, Dongmei Zhang, Xiancheng Chen, Li Yang, Yuquan Wei, Xia Zhao

**Affiliations:** 1 Department of Gynecology and Obstetrics, West China Second Hospital, Sichuan University, Chengdu, Sichuan, People's Republic of China; 2 State Key Laboratory Biotherapy and Cancer Center, West China Hospital, Chengdu, Sichuan, People's Republic of China; Ghent University, Belgium

## Abstract

Endostatin is an important endogenous inhibitor of neovascularization that has been widely used in anti-angiogenesis therapy for the treatment of cancer. However, its clinical application is largely hampered by its low efficacy. Human placenta-derived mesenchymal stem cells (hpMSCs) are particularly attractive cells for clinical use in cell-based therapies. In the present study, hpMSCs were isolated and characterized. We then evaluated the tumor targeting properties and antitumor effects of hpMSCs as gene delivery vehicles for ovarian cancer therapy. We efficiently engineered hpMSCs to deliver endostatin via adenoviral transduction mediated by Lipofectamine 2000. The tropism capacity of the engineered hpMSCs toward tumor cells was then confirmed by *in vitro* migration assays and *in vivo* by intraperitoneal injection of hpMSCs into nude mice. The hpMSCs expressing the human endostatin gene demonstrated preferential homing to the tumor site and significantly decreased the tumor volume without apparent systemic toxic effects. These observations were associated with significantly decreased blood sprouts and tumor cell proliferation as well as a dramatically increased tumor apoptosis index. These results suggested that hpMSCs are potentially an effective delivery vehicle for therapeutic genes for the treatment of ovarian cancer.

## Introduction

Ovarian cancer is one of the most common gynecologic malignancies, and it remains the fourth leading cause of cancer-related death among women [1]. Despite the many advances in surgical management, chemotherapy, and radiation therapy over the past decades, the prognosis for patients with advanced ovarian cancer remains poor, with a 5-year survival rate of less than 30% for patients with distant metastases. This low survival rate is primarily due to eventual tumor recurrence and emergence of drug-resistance [2]. Consequently, novel therapeutic approaches are urgently needed to change the future outlook of patients with ovarian cancer.

Angiogenesis plays a crucial role in the biological and pathological processes of cancer. Multiple lines of evidence have demonstrated that the growth and progression of most solid cancers are angiogenesis dependent and tumor angiogenesis is highly orchestrated by a balance between positive and negative regulators [3], [4]. To date, a large number of anti-angiogenesis agents have been identified. Endostatin, a 20-kDa carboxyl terminal fragment of the α1 chain of collagen XVIII that inhibits endothelial cell migration, proliferation, and induces apoptosis of vascular endothelial cells, has been considered as the most potent inhibitor of angiogenesis. It is well established that endostatin can effectively inhibit various solid tumors, such as small Lewis lung cell carcinoma [5], colon cancer [6], human breast cancer [7], hepatocellular carcinoma [8], [9], ovarian cancer [10], [11], and malignant melanoma [12]. However, as a protein drug, endostatin has a short half-life *in vivo* and easily loses its efficacy. Furthermore, the requirement for a frequent dosage regimen and high doses of expensive purified protein hampers its future clinical application. To overcome these shortcomings, the application of gene therapy has been explored. However, the gene delivery efficiency of plasmid vectors is very poor, and they also produce very low expression of endostatin. Other strategies have tried to overcome some of these issues in attempts to prolong the expression of endostatin. Adenovirus is considered one of the most efficient gene vectors and has been shown to generate high expression of endostatin for several days [4]. Nevertheless, limitations arise from the relatively short survival time of the virus, and these vectors cannot migrate specially to the tumor site and thus require location injection. Therefore, new and more effective therapeutic tools are needed that specifically target endostatin expression to the tumor cells.

Mesenchymal stem cells (MSCs) are multipotent stem cells with the ability to differentiate into a variety of cell types, including chondrocytes, osteoblasts, adipocytes, muscles, neurons, stromal cells, and other cell types [13]. Several studies have indicated that human placenta-derived mesenchymal stem cells (hpMSCs) are similar to stem cells from bone marrow with respect to cell characteristics and their potential for multilineage differentiation [14]–[16]. As placental tissues originate during the first stages of embryological development, these tissues might contain cells that have retained the prosperities of early embryonic cells from which they derive. Furthermore, the placenta is fundamental for maintaining fetomaternal tolerance during pregnancy, suggesting that cells present in placenta tissue may have immonomodulatory characteristics. Meanwhile, recent studies showed that mesenchymal isolated from placenta tissue have the ability to specifically homing to multiple tumor site. These three key aspects make cell from placenta extremely attractive candidate for possible use in cell therapy approaches in direct cancer therapy [17]. Some studies have engineered MSCs to express interferon β (IFNβ) in gliomas [18], metastatic melanoma [19], and breast cancer models [20]. MSC-delivered IFNβ has been shown to suppress tumor cell growth by inducing cancer cell differentiation and apoptosis, resulting in increased survival in these models [18], [19]. These studies showed the relevant molecular mechanism mediating cross-repressive interaction between engineered MSCs and tumor growth may involved in multiple aspects including: a. MSCs can travel to the same homing destination as the migrating cancer stem cell with unusual abilities to migrate to oncogenetic site; b. the cytokine hidden in the MSCs can evade the immune system surveillance and be well tolerated by the host without inducing an unacceptable immune response; c. viral-transduced MSCs can deliver viral initiatively to tumor sites and also increase the local viral dose by continuous viral replication and amplification [21]. These results showed MSCs could serve as potential candidate of vector for gene therapy. Meanwhile, hpMSCs have been shown to be more advantageous in cell procurement, storage, and transplantation than bone marrow-derived MSCs. Moreover, mesenchymal stromal cells from bone marrow have a risk of viral infection [22] and their differentiation capacity decreases significantly with donor age [23]. Furthermore, the placenta is generally discarded after birth; as such, this tissue is available in large supply, and the isolation of stem cell from this tissue does not involve any invasive procedures for the donor and avoids ethical controversy. These key aspects make cells isolated from placenta good candidates for possible use in cell therapy. The primary interest of this study has been focused on whether endostatin-engineered hpMSCs could specifically migrate to the tumor site and overcome tumor progression and metastases in a human ovarian cancer model.

## Materials and Methods

### Recombinant adenoviral vector construction

Construction of the recombinant endostatin adenovirus has been described in a previous study [10]. Briefly, the full-length human endostatin cDNA (about 570 bp) was amplified by RT-PCR. After sequence confirmation, the cDNA was cloned into a shuttle vector for the rescue of the recombinant E1-/E3-deleted adenovirus (AdEasy system) [24]. The viral particles were amplified in 293 cells, purified by two-step cesium chloride (CsCl) gradient ultracentrifugation, and measured by absorption at 260 nm. The virus titer was quantified using a standard TCID50 assay.

### Cell culture and reagents

The A2780s cell line and human embryonic kidney (HEK293) cells were obtained from ATCC (American Type Culture Collection, Manassas, VA, USA) and cultured in RPMI-1640 (Invitrogen, Carlsbad, CA, USA) routinely supplemented with 10% heat-inactivated fetal bovine serum plus ampicillin and streptomycin in a humidified 5% CO_2_ incubator at 37°C. Female nude mice (6–8 weeks old) were purchased from the Laboratory Animal Center of Sichuan University.

### hpMSC isolation and culture

Full-term human placenta was obtained from a healthy mother after donor's written consent under a tissue collection protocol approved by the institution's Institutional Review Board (IRB) of the West China Second Hospital. The informed consents were written down and all the documents were kept in West China Second Hospital's IRB. With sterile procedures, fetal deciduas placental tissue was cut into pieces of approximately 1 cm^3^ in size, washed with PBS, and digested with 1mg/ml I type collagenase (Sigma-Aldrich, St. Louis, MO, USA) at 37°C for 2 h. The cells were then separated via centrifugation over Ficoll–Hypaque separation medium at a 1.088 g/cm^3^ density gradient to remove unwanted cells. The collected cells were resuspended for culture in the L-DMEM medium (Sigma-Aldrich, St. Louis, MO, USA) supplemented with 20% FBS (Invitrogen, Carlsbad, CA, USA), 10 U basic fibroblast growth factor (Sigma-Aldrich, St. Louis, MO, USA), 2 mmol/l L-glutamine (Sigma-Aldrich, St. Louis, MO, USA), 100 U/mL penicillin (Sigma-Aldrich, St. Louis, MO, USA), and 100 μg/mL streptomycin (Sigma-Aldrich, St. Louis, MO, USA). The cells were then seeded in 75 cm^2^ flasks and cultured in a 37°C, 5% CO_2_ incubator with saturated humidity. After 48 h, non-adherent cells were removed by replacing the culture medium. Fresh medium was exchanged every 3 to 4 days. The cells were harvested by trypsinization (0.25% trypsin with 0.1% EDTA), subsequently passaged, and then used during the fourth passage for experiments.

### Cell surface phenotype and multipotent differential estimation

To distinguish hpMSCs, phenotypic characterization of CD29, CD44, CD105, CD73, CD166, CD90, CD34 and CD 45 were analyzed using a flow cytometer (Coulter EPICS Elite ESP) [25]. Cells were digested and incubated with antibody for 25 minutes at 4°C. Unstained, nonincubated cells served as control. Then cells were fixed in 4% buffered formalin and analyzed with a flow cytometer. Minimums of cells for 8,000-gated events were acquired for each sample. For osteogenic and adipogenic differentiation, hpMSCs were cultured at 5×10^4^ cells per well of 12-well plates in OsteoDiff or Adipodiff induction medium (Miltenyi Biotec GmBH) for 3 weeks, then Alizarin Red S and Oil-Red-O were utilized to visualize calcium deposits and fat droplets separately. To determined whether transfection with adenovirus would affect the multipotent characteristics, 48 hours after transfected with adenovirus, cells were cultured in OsteoDiff or Adipodiff induction medium for 3 weeks, then Oil-Red-O and Alizarin Red S were utilized to visualize calcium deposits and fat droplets respectively.

### hpMSC transfection and protein expression assays

The isolated MSC population with positive phenotype was expanded continuously for weeks until the plate-adhering hpMSCs reached >90% confluence and possessed sufficient anchorage activity and stability to endure the stresses of viral vector infection at a high multiplicity (usually between population doublings 4 and 7). Before transduction, the growth medium was removed and the cells were washed once with serum-free DMEM. Ad-hEndo-GFP was transfected using Lipofectamine 2000 (Invitrogen, Carlsbad, CA, USA) at a multiplicity of infection (MOI; viruses/cell) of 2000 (selected as the optimum among the MOIs of 1000, 2000, and 3000). After 48 h, vector and mock-transduced cells were analyzed under an inverted microscope (Axiovert 200; Carl Zeiss MicroImaging, Thornwood, NY, USA) and a flow cytometer was applied for evaluation of green fluorescence.

The cells transfected with Ad-hEndo (hpMSC-Ad-hEndo) and the control adenovirus (Ad-null) at an MOI of 2000 (2.5*10^8^ p.f.u/10^6^ cells in 1.0 ml of complete media) was then obtained, and the supernatant was harvested, concentrated by ultrafiltration (Centricon YM-3; Millipore, Bedford, MA, USA), and analyzed by Western blot. Proteins were separated using a 12% SDS-PAGE gel and transblotted onto a PVDF membrane (Bio-Rad, Hercules, CA, USA). The membrane was then blocked in 5% nonfat milk in 0.1% Tris-buffered saline with Tween 20 (TBST) for 1 h at room temperature and later probed with a rabbit polyclonal anti-endostatin antibody (Santa Cruz Biotechnology, Santa Cruz, CA, USA) diluted 1∶400 at 4°C overnight. Subsequently, the blots were incubated with a horseradish peroxidase-conjugated anti-rabbit immunoglobulin (1∶5000; Sigma-Aldrich, St Louis, MO, USA) for 1 h at room temperature. The protein bands were detected using an ECL detection kit (Sigma-Aldrich, St. Louis, MO, USA).

Furthermore, we also performed ELISA assay to determine the expression level of endostatin secreted by engineered hpMSCs *in vitro*. As described before, the isolated hpMSCs were transfected with adenovirus carrying endostatin, and on 48, 72 and 96 hours post transfection, the supernatants were collected and assayed for human endostatin gene expression levels using a Human Endostatin Quantikine ELISA Kit (R&D Systems).

### *In vitro* hpMSC migration assay

A2780 (1*10^6^) or HEK293 (5*10^5^) cells were seeded in 500 μl of medium/well in 24-well plates. After 24 hours, a Millicell insert (8 µm pore size; Millipore, Billerica, MA, USA) was placed above the A2780 or HT293 cell layer, and hpMSCs cells were plated in the upper well and cultured for 48 h. Due to the polycarbonate membrane of the Millicell, the cells shared the same medium with no direct cell-cell interaction between the chambers. The membrane was then washed three times with PBS. The hpMSCs on the lower side of the membrane were dyed with crystal violet, and five random fields were counted using an inverted microscope (Axiovert 200; Carl Zeiss MicroImaging, Thornwood, NY, USA). These experiments were conducted in triplicate.

### Determination of hpMSC homing to the tumor site

Tumors were established in mice by intraperitoneal (i.p.) injection of 5*10^6^ A2780s cells. After 14 days of tumor challenge when tumor nodules were palpable, hpMSCs were transfected with Ad-GFP using Lipofectamine as described before. After 48 h, the cells were collected, washed once with PBS and suspended in fresh medium. Then cells were injected i.p. at 2*10^5^ cells per injection. Non-tumor bearing mice were applied as control. Three days and 2 weeks after injection, tumor nodules and orangs (heart, liver, spleen, lung, kidney) were collected and frozen in Optimal Cutting Temperature compound (OCT) respectively. Fluorescent hpMSCs were detected in fresh cryosections (3–5 µm) of the tumor samples and organs using a fluorescent microscope.

### Treatment of the experimental xenograft model

Female nude BALB/c mice 6–8 weeks old were purchased from the experimental animal center of Sichuan University. The research protocol was reviewed and approved by the Institutional Animal Care and Treatment Committee of Sichuan University. Human ovarian cancer was established by i.p. injection of nude mice with 5*10^6^ A2780 cells in 200 µl PBS. Before i.p. administration, hpMSCs were transfected with Ad-hEndo or Ad-null at an MOI of 2000 as described above. Mice were randomly divided into four groups (n = 5 animals/group): (a) mice treated with 0.9% normal saline (NS); (b) mice treated with hpMSCs at 2*10^5^/mouse; (c) mice treated with Ad-null–transfected hpMSCs at 2*10^5^/mouse; (d) mice treated with Ad-hEndo–transfected hpMSCs at 2*10^5^/mouse. Treatment started 5 days after tumor-cell challenge and was administrated via i.p. injection every 3 days for a total of 6 times. The mice were monitored on a daily basis for tumor burden, abdominal distension, cachexia, and other abnormalities. The mice were sacrificed 1 week after the last administration, and the i.p. tumors were then excised and weighted.

### Quantitation of cell proliferation and angiogenic microvessel density

Primary tumor samples with adjacent tissues and relevant internal organs were harvested, fixed in 4% paraformaldehyde, and then embedded in paraffin. For immunohistochemistry analysis, sections 3–5 μm thick were cut from the paraffin blocks, deparaffinized in xylene, and rehydrated with a series of graded EtOH. Antigen retrieval was carried out by autoclaving the sections in retrieval buffer (10 mmol/l EDTA citrate buffer [pH 6.0]; Dako, Glostrup, Denmark) for 4 min in saturated steam after up-pressure gaining (126°C, 1.6 bar, 23 psi). Endogenous peroxidase activity was blocked with 3% hydrogen peroxide at room temperature for 10 min free of light, and nonspecific binding of reagents was quenched by incubation of the sections for 20 min in 10% normal goat or rabbit serum. The sections were then incubated with goat anti-human Ki-67 polyclonal antibodies (1∶150; Maixin-Bio, Fuzhou, China) and polyclonal rat anti-human CD31 (1∶200; Maixin-Bio, Fuzhou, China) overnight in a moist chamber at 4°C; this was followed by incubation with biotinylated rabbit anti-goat/rat IgG and then streptavidin–biotin–horseradish peroxidase complex at 37°C for 45 min. The immunoreactions were finally developed (visualized with diaminobenzidine [DAB] solution as a chromogen) using a DAKO Liquid DAB+ substrate-chromogen system, and the crimson yellow precipitates were identified as positive staining. Counterstaining was gently performed with ameliorative Gill's hematoxylin, and the slides were dehydrated and mounted with glass coverslips. The sections were then visualized using a microscope at 400x magnification (Olympus, Tokyo, Japan). The Ki-67-positive cells or blood vessels were counted from three areas in each section in a blinded manner.

### Alginate encapsulation assay

Alginate bead containing tumor cell assay was described in details previously [26]. Briefly, cultured A2780 cells were re-suspended with 1.5% (m/v) sodium alginate (Sigma-Aldrich, St. Louis, MO, USA), and then the tumor cell alginate solution was dropped into a swirling bath of 0.25 M CaCl2 in order to form droplets containing about 1×10^5^ tumor cells per bead. After anesthetized, the nude mice were implanted s.c. with four beads into an incision on the back, the incisions were sutured with surgical clamps. The hpMSCs-Ad-hEndo (at an MOI of 2000) was injected i.p. on day 3, 6, 9, 12 after bead implantation, with hpMSCs-Ad-null, hpMSCs or saline as control. At 14 days, the mice were injected i.v. with 100 μl FITC-dex- tran solution (Sigma-Aldrich, St. Louis, MO, USA) (100 mg/kg) and were sacrificed 20 minutes later. Image of the alginate implants was taken by using SPOT FIEX camera. Alginate beads were transferred to tubes containing 2 ml saline. The tubes were mixed by a vortex for 20 s and centrifuged (3 min; 1000×g). Finally the fluorescence of the supernatant was measured to quantify blood vessel formation.

### Analysis of apoptosis in tumor tissues

Apoptosis analysis was performed by means of TUNEL (terminal deoxynucleotidyl transferase-mediated dUTP nick-end labeling) using a DeadEnd Fluorometric TUNEL System (Promega, Madison, WI, USA) according to the manufacturer's protocol. TUNEL-positive nuclei, pyknotic nuclei with dark green fluorescent staining, were visualized and analyzed under a fluorescence microscope (Olympus, Tokyo, Japan). The apoptosis index was determined by two dependent investigators by counting TUNEL-positive cells in five high power fields per slide.

### Evaluation of potential toxicity

In order to evaluate the potential side effects and toxicity of hpMSCs, the relevant indexes such as weight loss, anorexia, diarrhea, cachexia, skin ulceration, ruffled fur, and toxic death were observed. After sacrifice of the mice, various organs (heart, liver, spleen, lung, kidney etc.) were harvested and fixed in 4% paraformaldehyde in PBS. Sections of 3–5 µm were stained with hematoxylin and eosin (H&E) and observed under a microscope by two pathologists in a blinded manner.

### Statistical analysis

The statistical differences in tumor weight, animal weight, percent apoptosis, and microvessel density were determined using one-way analysis of variance (ANOVA). A P value <0.05 was considered statistically significant.

## Results

### Phenotype analysis and multipotent characteristics of isolated cells

**Figure 1:**
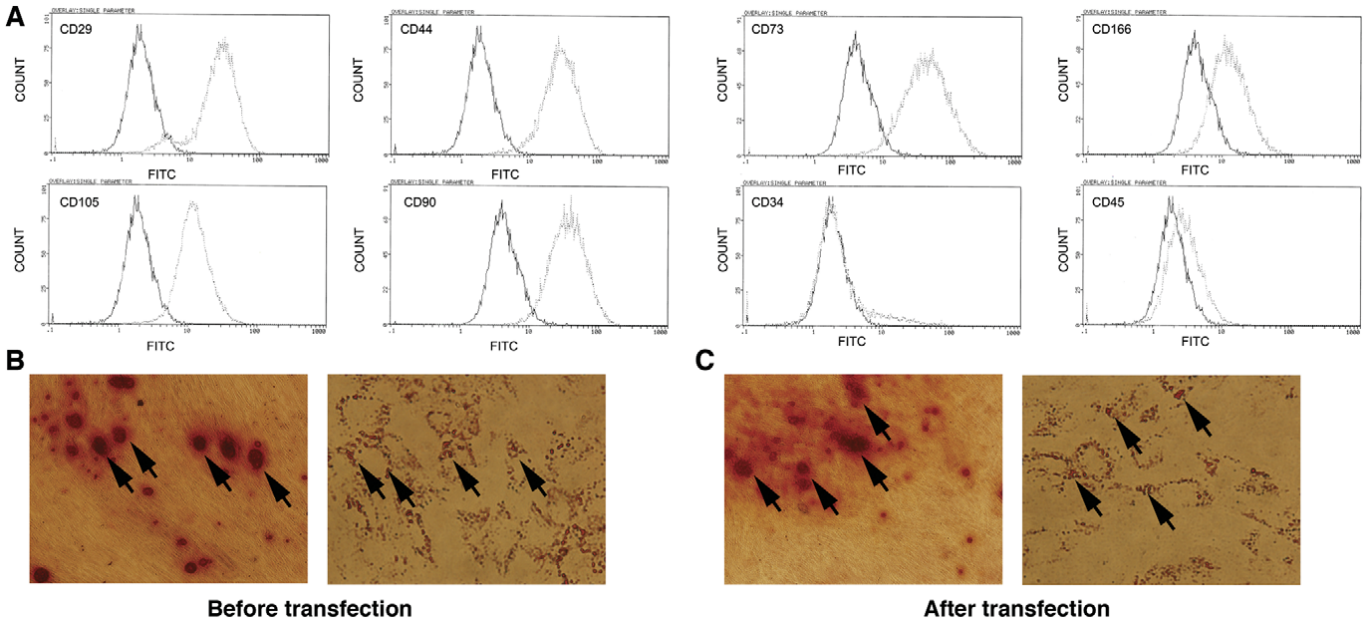
Analyze of the purified hpMSCs population. A. The phenotype characteristics of purified MSCs were verified by flow cytometry analysis, which showed that these expanded and plate-adhering populations of cells (at doublings 3 and 5) were positive for CD29 (>95%), CD44 (>95%), CD73 (>99%), CD90 (>95%), CD166 (>95%) and CD105 (>95%) surface marker expression and negative for CD34 (<3%) and CD45 (<3%). The solid line denotes unstained control cells and dotted line denotes isolated mesenchymal stem cells. B. Osteogenic and adipogenic differentiation determination in isolated mesenchymal stem cells. Left panel indicated positive calcium deposit (arrows), right panel indicated lipid vacuoles (arrows). C. Osteogenic and adipogenic differentiation determination in isolated mesenchymal stem cells after transfected with adenovirus. 48 h after transfection, cells were washed with PBS and cultured in OsteoDiff or Adipodiff induction medium for 3 weeks, then Oil-Red-O and Alizarin Red S were utilized to visualize calcium deposits and fat droplets separately. Lipid vacuoles and calcium deposits (arrows) could be visualized (×100).

Flow cytometry analyses indicated that the population of plate-adhering cells was positive for mesenchymal stem cell markers CD29 (>95%), CD44 (>95%), CD73 (>99%), CD90 (>95%), CD166 (>95%), CD105 (>95%) and negative for hematopietic markers CD34 (<3%) and CD45 (<3%), which was consistent with the characteristics of MSCs (Figure 1A). The general definition of MSCs includes both phenotypic and functional criteria, so we applied the osteogenic and adipogenic differentiation assay to indentify the multipotent characteristics before and after adenovirus transfection. For osteogenic and adipogenic differentiation, hpMSCs were cultured at 5×10^4^ cells per well of 12-well plates in OsteoDiff or Adipodiff induction medium (Miltenyi Biotec GmBH) for 3 weeks, then Alizarin Red S and Oil-Red-O were utilized to visualize calcium deposits and fat droplets separately. As showed in Figure 1B, 1C, visualizing lipid vacuoles and calcium deposits pointing to a broader multipotent differentiation capacity of our hpMSCs. We also determined whether transfection with adenovirus would affect the multipotent characteristics. 48 hours after transfected with adenovirus, cells were cultured in OsteoDiff or Adipodiff induction medium for 3 weeks, then Oil-Red-O and Alizarin Red S were utilized to visualize calcium deposits and fat droplets separately. As showed in Figure 1C, positive lipid vacuoles and calcium deposits could be visualized.

### *In vitro* expression of hpMSC-Ad-hEndo

**Figure 2:**
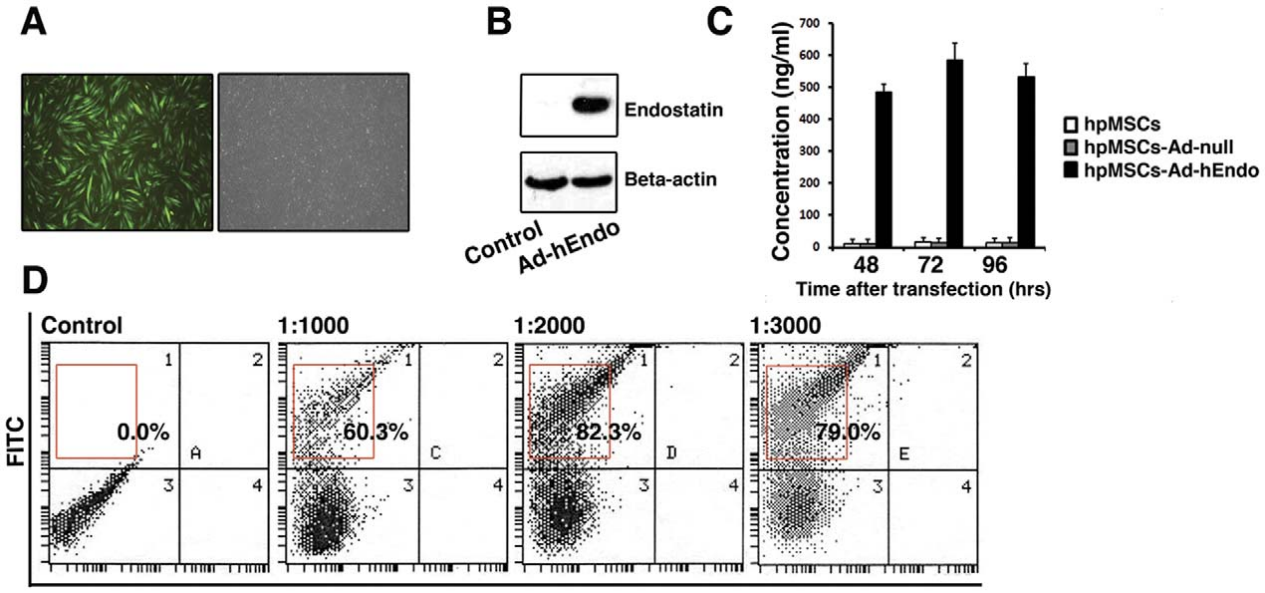
Fluorescence images of transduced hpMSCs and the expression of endostatin protein determined by ELISA and Western blots. A. GFP fluorescence was assessed in the FITC channel. The hpMSCs transfected with GFP-labeled adenovirus showed green. Original magnification×400. B. Cells transfected with Ad-hEndo and control adenovirus (Ad-null) at an MOI of 2000 were analyzed by Western blot to detect the expression of the endostatin protein. hpMSCs transfected with Ad-hEndo showed obvious expression of endostatin protein when compared with control. C. To verify the expression level of endostatin secreted by engineered hpMSCs *in vitro*, ELISA assay was performed to evaluate endostatin in the supernatants of cells transfected with adenovirus carrying endostatin, hpMSCs and hpMSCs transfected with Ad-null were applied as control. At 48 h, 72 h and 96 h post transfection, cell culture supernatants were collected and endostatin concentration were determined. The concentration of endostatin in the cell culture supernatants was significantly higher than the control groups and the level of endotatin also increased with time and remained at a high level 96 h after transfection. D. Flow cytometry was applied to evaluate the transfection efficiency of Ad-hEndo-GFP. The transfection rate at MOI 1000, 2000 AND 3000 was 60.3%, 82.3% and 79% respectively. Compared with the control, infection at an MOI of 2000 resulted in higher transfection efficiency. The red frame represents positive zone on each plot.

Since human placenta-derived MSCs are relatively resistant to wild-type adenoviral infection due to their low expression of the adenoviral receptor CAR, we conducted Lipofectamine 2000-mediated adenoviral transduction. The gene delivery efficiency of the adenovirus into hpMSCs was determined by microscope evaluation. We found that after transduction with the recombinant Ad-hEndo-GFP at an MOI of 2000 and 3000, almost all of the cells emitted green fluorescence following cultivation under plate-adhering conditions (Figure 2A). However, along with increased infection multiplicity, cell death was also augmented, possibly reflecting cell damage due to excessive transgene expression. Furthermore, flow cytometry was used to measure the transduction efficiency. The findings indicated that it was efficient at an MOI of 2000, as approximately 82.3% of hpMSC-hEndo cells consistently expressed GFP at this MOI (Figure 2D). Taken together, we chose to use cells engineered under these transduction conditions (an MOI of 2000) in all subsequent experiments. Subsequently, to verify whether the endostatin protein was secreted from hpMSC-Ad-hEndo cells, Western blot analysis was performed. As showed in Figure 2B, at an MOI of 2000, a distinct band of about 20 kDa, corresponding to the size of endostatin, was expressed in the Ad-hEndo-treated cells but not in Ad-null treated cells. Furthermore, we also performed ELISA assay to detect endostatin secreted from hpMSC-Ad-hEndo. As showed in Figure 2C, the concentration of endostatin in the cell culture supernatants was significantly higher than the control groups and the level of endotatin also increased with time and remained at a high level 96 h after transfection. This assay verifies that endostatin can be secreted efficiently from the engineered hpMSCs.

### The migratory capacity of hpMSCs toward tumor cells *in vitro* and *in vivo*

**Figure 3:**
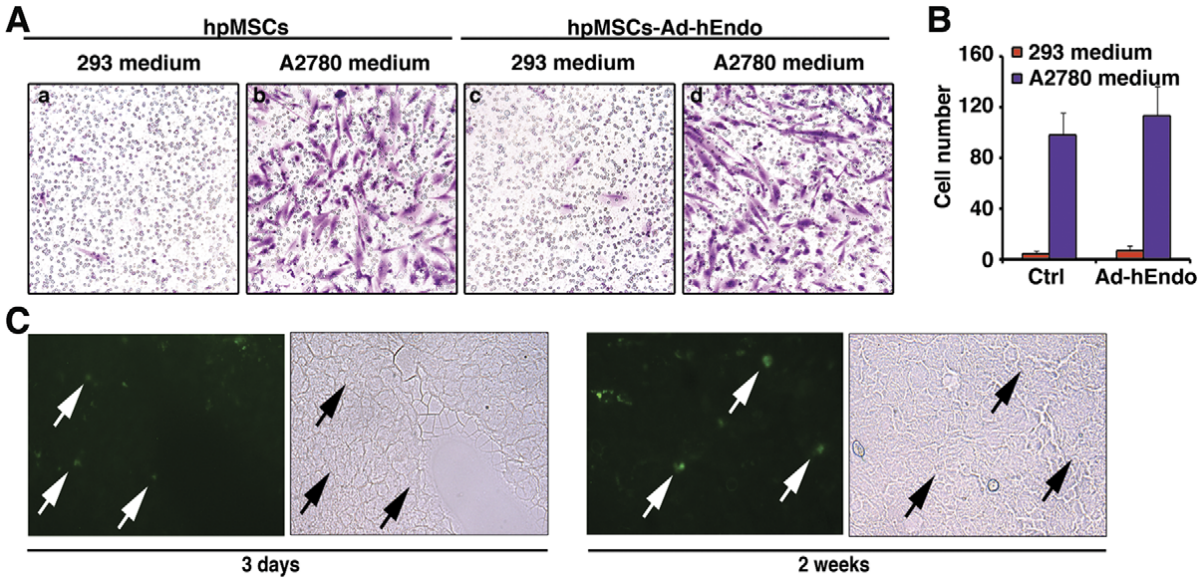
Migratory ability of transduced hpMSCs *in vitro* and *in vivo*. A. Migration of hpMSCs/hpMSCs-Ad-Endo cells through an 8-um Millicell membrane. HpMSCs were cultured in 24-well plate onto 8-um pore-sized membranes, and A2780s or 293 cells were cultured in the lower chamber. The cells migrated to the lower side of the membrane were labeled with methyl violet. Original magnification×400. B. Induction of hpMSCs/hpMSCs-Ad-Endo migration stimulated by A2780s or 293 cells. Compared with 293 cells, A2780s cells promoted migration of hpMSCs//hpMSCs-Ad-Endo significantly. Data shown as means ± SD. C. To verify the specific homing property of transducted mesenchymal to tumor site, we transducted hpMSCs with adenovirus carrying GFP and injected i.p. to nude mice with established peritoneal ovarian tumor. After 3 days and 2 weeks of i.p. injection, mice were sacrificed and tumor nodules and organs were collected. Left panel: 3 days after i.p. injection, GFP signal (arrow) were detected at tumor periphery. Right panel: 2 weeks after i.p. injection, positive GFP signal were detected in the tumor parenchymal (arrow) which indicated hpMSCs persist in the xenograft and can integrate into tumor focus. There is no positive GFP signal detected in the organs.

It has been proposed that factors released from cancer cells may be potential chemoattractants involved in the tropism of MSCs. To evaluate the migratory capacity of hpMSCs toward A2780 cells, *in vitro* migration assays using Millicell inserts were conducted. Conditioned medium from 293 cells was used as a control. Only a few hpMSCs-Ad-hEndo cells migrated toward the 293-conditioned medium, whereas the migration of hpMSCs-Ad-hEndo was significantly stimulated by conditioned medium from the human ovarian cancer A2780 cells (P<0.01; Figure 3A). When the numbers of migrated cells on the underside of the Millicell inserts were calculated, it was confirmed that unmodified hpMSCs migrated to the conditioned medium from A2780 cells in a similar pattern as that of hpMSCs-Ad-hEndo. Together, our data indicated that under the conditions described, hpMSCs showed significant migratory capacity and A2780 cells possessed a greater capacity for inducing migration of MSCs compared with 293 cells (P<0.05; Figure 3B). To determine if hpMSCs preferentially homed to human ovarian xenografts, we established nude mice peritoneal ovarian cancer model. 2 weeks after i.p. injection of A2780 cells and tumors were palpable, we i.p. injected mesenchymal stem cells transfected with adenovirus carrying GFP, and non-tumor bearing mice were used as control. At the time point of 3 days and 2 weeks after i.p. injection of transfected mesenchymal stem cells, mice were sacrificed and tumor nodules, liver, spleen, lung, kidney and heart were collected and GFP signal was measured. As showed in the Figure 3C, 3 days after i.p. injection, GFP signals were detected at tumor periphery. 2 weeks after i.p. injection, interestingly we detected positive signal in the tumor parenchymal. This finding suggested that the injected mesenchymal stem cells persist in the xenograft and can integrate into tumor focus. Furthermore, there is no positive signal found in the organs of tumor bearing and non-tumor bearing mice.

### Engineered hpMSCs exert an antitumor effect on tumor growth *in vivo*

**Figure 4:**
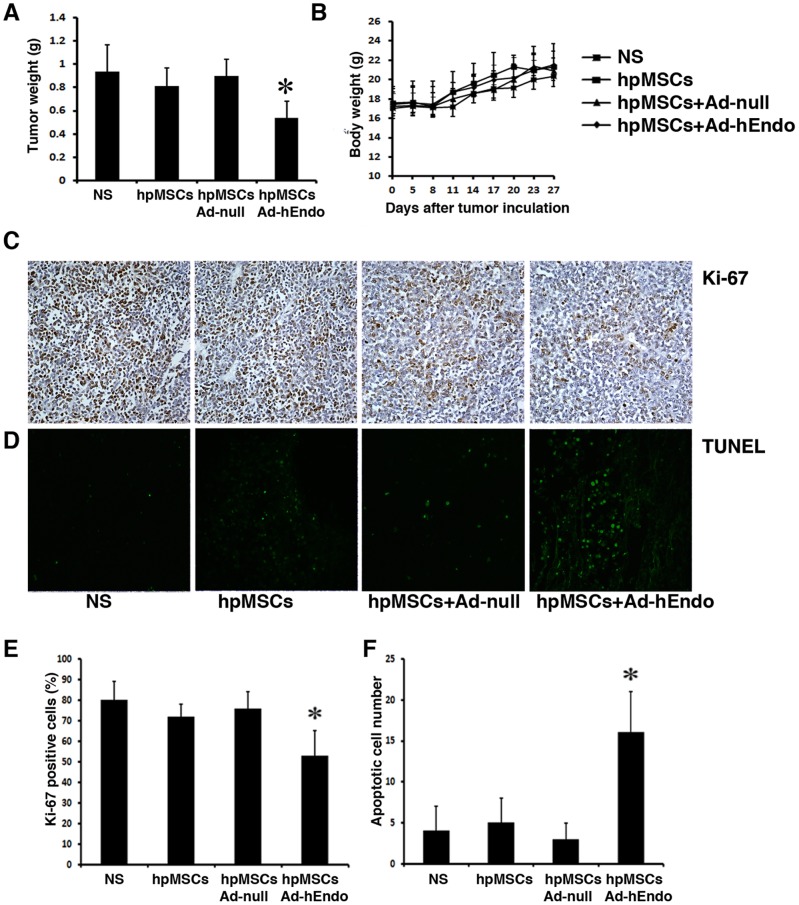
Tumor suppression effect following treatment with hpMSCs. A. The antitumor effects of hpMSCs + Ad-hEndo versus hpMSCs, hpMSCs + Ad-null, and normal saline (NS). The tumor-bearing mice in each group received the corresponding treatment as detailed in the materials and methods. hpMSCs + Ad-hEndo showed significant differences compared with the controls (hpMSCs, hpMSCs + Ad-null, and NS; P<0.05) B. Lack of toxicity-dependent body weight loss in tumor-bearing mice treated with hpMSCs + Ad-hEndo. There was no statistical difference between the groups (P>0.05). Data are shown as the means ± SD. C. Analysis of cell proliferation via Ki-67 staining. Ki-67–positive cells were abundant in the NS, hpMSCs, and hpMSCs + Ad-null groups; whereas, the hpMSCs + Ad-hEndo group demonstrated substantially decreased Ki-67–positive expression. Original magnification×400. D. Analysis of apoptosis via TUNEL analysis. The hpMSCs + Ad-hEndo group demonstrated a significant enhancement of TUNEL-positive nuclei. No appreciable difference was observed among the NS, hpMSCs, and hpMSCs + Ad-null groups. Original magnification×400. E. Percentages of Ki-67-positive nuclei showed tremendous reduction happened in hpMSCs-Ad-hEndo group. (*, P<0.05) F. hpMSCs-Ad-hEndo markedly increased the percent apoptosis versus controls. Values are presented as means ± SD. (*, P<0.05)

In this study, an A2780 xenograft model by i.p. injection was established to analyze the therapeutic potential of hpMSCs-Ad-hEndo. When small tumor nodules were palpable after approximately 5 days of peritoneal tumor challenge, nude mice were randomly divided to received administration of 0.9% NS, hpMSCs, hpMSC-Ad-null, or hpMSC-Ad-hEndo. All mice were treated every 3 days for a total of 6 times. The results showed that the treatment with hpMSCs-Ad-hEndo resulted in a significant regression of the established tumors compared with the NS, hpMSCs, and hpMSCs-Ad-null treatment groups (P<0.05; Figure 4A). Tumor sections from each group were stained with Ki67 antibody (Figure 4C) to determine the level of cell proliferation. Tumors of the control groups, including NS, hpMSCs, and hpMSCs-Ad-null, showed high levels of cell proliferation. However, those submitted to hpMSCs-Ad-hEndo treatment showed decreased cell proliferation. There was no difference in cell proliferation between the controls, but decreased cell proliferation was seen in the hpMSCs-Ad-hEndo group (P<0.05; Figure 4E). To further explore the role of hpMSCs-Ad-hEndo therapy in tumors *in vivo*, TUNEL assays were carried out to detect apoptosis within the tumor tissues. Cell nuclei stained with dark green, as viewed by fluorescence microcopy (400× magnification), indicated apoptosis, and these cells were recorded as having TUNEL-positive nuclei. The number of cells was counted in five random fields, avoiding areas of necrosis. The results revealed many TUNEL-positive nuclei in the hpMSCs-Ad-hEndo–treated tumor tissues, whereas such nuclei were rare in tumor tissues of the control groups (Figure 4D), suggesting that engineered hpMSCs-Ad-hEndo had a significant induction effect on tumor cell apoptosis *in vivo* (P<0.05; Figure 4F).

**Figure 5:**
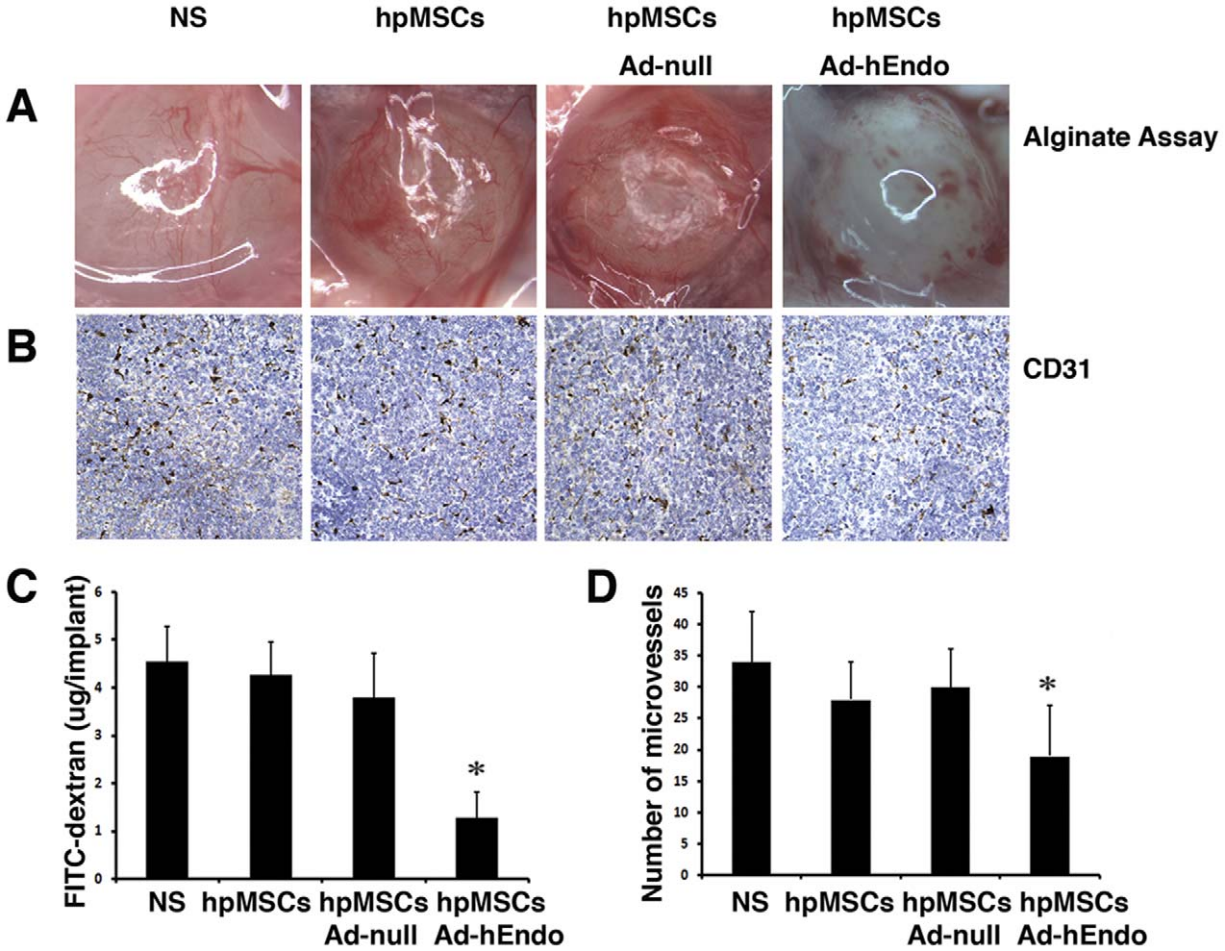
Evaluation of anti-angiogenesis effect by alginate assay and CD31 immunohistochemistry. **A**. Anti-angiogenesis of hpMSCs + Ad-hEndo versus hpMSCs, hpMSCs + Ad-null, and normal saline (NS). Alginate beads containing A2780s tumor cells were implanted subcutaneously into nude mice. The hpMSCs-Ad-hEndo (1×10^9^pfu/100 μl) was injected i.p. on day 3, 6, 9, 12 after bead implantation, with hpMSCs-Ad-null, hpMSCs or saline as control. At day 14, image of the alginate implants was taken and indicated decreased vascularization in beads treated with hpMSCs-Ad-hEndo. B. Analysis of angiogenesis among the groups via CD31 staining. CD31-positive cells were plentiful in the NS, hpMSCs, and hpMSCs + Ad-null groups; however, few positive cells were found in the hpMSCs + Ad-hEndo group. C. FITC-dextran uptake decrease showed the reduction of vascularization in beads treated with hpMSCs-Ad-hEndo. (*, P<0.05) D. Vessel density was determined by counting the number of microvessels per high-power field (×400) and showed hpMSCs-Ad-hEndo significantly reduced CD31 positive microvessels compared with controls. Values are presented as means ± SD. (*, P<0.05)

The inhibition of angiogenesis in mice treated with hpMSCs expressing endostatin was further confirmed in alginate encapsulation assay. As shown in Figure 5A, the surface vessel densities were much lower in hpMSCs-Ad-hEndo group. The FITC-dextran uptake was apparently reduced in beads which receiving hpMSCs-Ad-hEndo treatment and there was no difference in groups treated with NS, hpMSCs and hpMSC-Ad-null (Figure 5C, P<0.05). Furthermore, we also performed immunohistochemistry to detect angiogenesis in tumor tissues, that is, tumor sections from each group were stained with anti-CD31 antibody (Figure 5B) to determine the level of microvessel density. Tumors of the control groups, including NS, hpMSCs, and hpMSCs-Ad-null, showed high densities of microvessels. However, those submitted to hpMSCs-Ad-hEndo treatment showed a low density of microvessels. Moreover, there was no difference in microvessel counts between the controls, but decreased microvessel density was seen in the hpMSCs-Ad-hEndo group (Figure 5D, P<0.05).

Animals treated 6 times with hpMSCs-Ad-hEndo were investigated for potential toxicity. The weight of mice was monitored every 3 days throughout the entire experiment and considered a parameter for evaluation of physical status, anorexia, and cachexia. Our result showed that there was no evidence of toxicity in terms of loss of body weight in the hpMSCs-Ad-hEndo group (Figure 4B). In addition, no conspicuous adverse effects in gross measures were observed, such as appetite, feeding, ruffling of fur, behavior, toxic death, etc. Furthermore, H&E histological staining of the heart, liver, spleen, lung and kidney investigated by two pathologists did not reveal any significant differences among the hpMSCs-Ad-hEndo–treated group and control groups.

## Discussion

It is known that angiogenesis plays a crucial role in biological and pathological processes involved in cancer. Multiple lines of evidence have demonstrated that the growth and progression of most solid cancers are angiogenesis dependent. Until now, the main means of endostatin therapy included protein, plasmid, and viral therapies. However, because of its short functional half-life, only large amounts of endostatin can promise successful therapy, and thus new therapeutic methods are urgently needed. In our study, we investigated the therapeutic potential of hpMSCs as an intermediate vector for an endostatin expressing-adenovirus for the treatment of human ovarian cancer.

Although the benefits of cells as viral vectors have been recognized, only a few practical attempts of using cells as viral carriers have been reported to date. The cell type used for delivery of the virus is a critical determinant of the efficacy of these treatments. As previously reported, MSCs that possess tumor-homing capacity are considered promising cell populations for cancer application. In our study, we successfully isolated hpMSCs from human term placenta and analyzed their phenotype using flow cytometry. Our results indicated that the purified hpMSCs (at doublings 3 and 5) were positive for CD29, CD44, CD73, CD90, CD166 and CD105 surface marker expression and negative for CD45 and CD34, which is in accordance with the criterion of MSCs.

In our study, we constructed hpMSCs encoding a human recombinant endostatin gene, and the transduction efficiency was measured by flow cytometry and transgene expression was detected by Western blot analysis and ELISA assay. These data indicated that the endostatin protein could be secreted from these cells. Furthermore, we applied an *in vitro* migration assay to clearly demonstrate the tumor tropism of these hpMSCs *in vitro*. Our data suggested that hpMSCs possessed great migratory capacity and that A2780s cells could significantly mediate the migration of hpMSCs, similar to inflammatory and damaged tissues that can induce the accumulation of hpMSCs. In contrast, 293 cells didn't show the same effect as A2780s cells. Though the precise mechanism behind the specific homing of hpMSCs to tumors is still difficult to pinpoint, prior research has indicated that the most likely cause of this phenomenon is due to a high local concentration of chemotactic gradients generated from the tumors. hpMSCs have a large variety of chemokine and cytokine receptors on their cell surface and respond functionally to these ligands *in vitro*, and manipulation of these receptors and ligands has been shown to alter the migration pattern of hpMSCs *in vivo* [27]–[30]. Tumors are also known to produce a large array of chemokines and cytokines, such as vascular endothelial cell growth factors, fibroblast growth factor, platelet derived growth factor [31], monocyte chemoattractant protein-1 [30], hypoxia-inducible factor [32], [33], and hepatocyte growth factor [34]. All of these factors could serve as ligands for the hpMSCs receptors.

Furthermore, we also evaluated the ability of these engineered hpMSCs to specifically target tumors *in vivo*. The extent of the tumor-homing ability of injected hpMSCs has not been previously investigated, and the tumor localization and proliferation of injected MSCs have only been confirmed in animal models of lung metastases [19] and glioma. It is possible that tumor cells can attract MSCs; however, the specificity of MSCs may be influenced by the experimental conditions, because in lung metastasis models the homing ability may be enhanced after systemic injection due to the mechanical trapping of these cells in the capillary system. In our study, we applied an i.p. model in the evaluation of the tropism ability of hpMSCs. The accessibility of tumor nodules in the peritoneal ovarian cancer model also gave us the opportunity to introduce the cell carriers locally, avoiding the cell trapping effect of systematic injection before tumor homing. We used hpMSCs transfected with Ad-GFP to show that hpMSCs preferentially localized to growing tumor nodules. We found that 3 days after i.p. injection, GFP signals were detected at tumor periphery and after 2 weeks positive signal can be detected in the tumor parenchymal. This finding suggested that the injected mesenchymal stem cells persist in the xenograft and can integrate into tumor focus. However, we only investigated the homing effect of hpMSCs in short time periods and did not follow the remote fate of the injected cells, because our strategy was to exploit only immediate routing of cells to tumors and delivery of oncolytic viruses does not rely on long-term survival and proliferation of the cell carriers.

In our *in vivo* treatment study, the engineered hpMSCs showed lasting inhibition toward established A2780s tumors, indicating the prolonged expression of endostatin. Furthermore, tumor cell proliferation and angiogenesis were clearly inhibited. The possible molecular mechanism associated with the anticarcinogenic effects of these cells may be associate with multiple actions, including (i) the ability of hpMSCs to incorporate into tumors, as stromal elements may allow these cells to release virus from the inside of the tumor; (ii) the released virus may be able to destroy tumor cells by viral replication, and following oncolysis newly produced virus would then be released into the surrounding tumor tissue; (iii) increased endostatin concentrations can inhibit preneoplastic neovascularization, depriving preclinical malignant cells of the nutrient uptake required for multiplication. Thus, we reasoned that hpMSCs represent an ideal population of virus carrier cells.

In conclusion, our investigation has revealed the profound antitumor and antimetastasis ability and low toxicity of hpMSCs-Ad-hEndo in a human ovarian cancer metastatic model. This approach strongly inhibited tumor microvessel formation and tumor cell proliferation, which could indicate a new and powerful therapeutic tool for the treatment of advanced human malignancies. Further studies are needed to elucidate the precise mechanisms of the antitumor activity of hpMSCs-Ad-hEndo. Our research suggested that hpMSCs-Ad-hEndo had a local anti-tumor ability and a strong anti-angiogenesis effect following i.p. injection.
